# Phenomenological Considerations of the World of the Obsessive Patient

**DOI:** 10.3390/jcm12134193

**Published:** 2023-06-21

**Authors:** Francesco Demaria, Maria Pontillo, Domenica Bellantoni, Cristina Di Vincenzo, Stefano Vicari

**Affiliations:** 1 Child and Adolescent Neuropsychiatry Unit, Department of Neuroscience, Bambino Gesù Children’s Hospital, IRCCS, 00146 Rome, Italy; maria.pontillo@opbg.net (M.P.); domenica.bellantoni@opbg.net (D.B.); cristina.divincenzo@opbg.net (C.D.V.); stefano.vicari@opbg.net (S.V.); 2 Department of Life Sciences and Public Health, Catholic University of the Sacred Heart, 00168 Rome, Italy

**Keywords:** obsessive patient, suffering, cognitive patterns, phenomenology

## Abstract

Despite significant scientific advances in research on obsessive-compulsive disorder (OCD), the psychological and behavioral symptoms of this pathological condition remain hard to understand, until they seem paradoxical. The present work seeks to consider the significance and potential contribution of a phenomenological reading of OCD and how phenomenalism has influenced some cognitive models of this disorder. Transcendental phenomenology is a philosophical approach that attaches primary importance to intuitive experience and considers all phenomena intrinsically associated with the subject’s inner world. Thus, the subject’s intuition is considered the starting point for understanding their essential experience. This approach has had a profound influence on modern cognitive sciences. Among current cognitive models, post-rationalist cognitivism and cognitive neuropsychological psychotherapy seem most effective in capturing the world experiences of OCD patients. Both apply a phenomenological approach to identify these experiences, which are typically characterized by hyper-reflexivity, at the expense of ‘natural evidence.’ The models have found that OCD patients experience the world emotionally as a sterile set of rules, and this experience determines their suffering.

## 1. Introduction

Obsessive-compulsive disorder (OCD) is a neuropsychiatric disorder characterized by the presence of obsessive (i.e., intrusive, repetitive, or unwanted) thoughts and compulsive behaviors or mental acts [1]. The disorder has a global prevalence of 0.25–3.0% [2]. However, obsessive-compulsive symptoms (OCS), more generally, are widespread. Among children and adolescents, OCS prevalence is reported in the range of 7–35% [3], and 30–50% of adults with OCD experience symptoms before the age of 18 [4]. OCD is a chronic life condition [5,6].

Obsessions and compulsions associated with OCD can be time-consuming (often involving more than 1 h per day) and may significantly interfere with everyday life habits, compromising quality of life [7,8]. In the DSM-5, OCD is recognized within the specific diagnostic category of obsessive-compulsive and related disorders; this deviates from its previous classification within anxiety disorders [9], reflecting scientific advances [10,11,12,13,14]. However, OCD remains a disorder with paradoxical psychological and behavioral symptoms [15,16] (i.e., ‘certain thoughts can lead to catastrophic events’, ‘thinking of doing something is equivalent to doing it’, and ‘not preventing something is equivalent to causing it’).

Bartoletti A. [17] also emphasizes the mechanism of “paradoxical repression” in order to explain the control that the patient with OCD must have over their obsessive thoughts/fixations. Individuals with OCD attempt not to think about and avoid unacceptable obsessions that torment them. However, thinking in order to not think means thinking more; thus, individuals who suffer from this disorder end up feeding their obsession and becoming completely trapped in this mechanism [18].

Thus, it requires further research into not only its clinical and behavioral aspects but also its experiential aspects [19,20].

Phenomenology contributes evidence of lived experience, constitutive corporeity, irreversible temporality, and intersubjectivity to the cognitive sciences. It does not consider the mind a mere organ affixed to and complementing a body, but an effect of the body’s operations in the world. In this way, it advances a radical and, at the same time, scientifically reliable proposal. Since its inception, it has maintained a lively dialogue with experimental psychology, attempting to define itself in contrast to it, while also accepting its demands. While the ‘descriptive’ psychopathology of OCD has been well-defined for some time [21], no satisfactory ‘explanatory’ psychopathological model has been advanced. Recent attempts to outline such a model have mainly drawn on cognitive therapy approaches. In contrast, the present work seeks to consider the meaning and potential significance of a phenomenological understanding of this pathology and how phenomenalism has influenced its cognitive reference models.

## 2. Research Objective

For a long time, clinicians have been curious about the main characteristics of patients suffering from OCD [19,20]. Studies have focused on the nature of their obsessions and the meaning of their distress, which they attempt to reduce via compulsions [22,23]; as well as the perception of disgust [24,25,26] and its pervasiveness [7,8]. However, to date, descriptive research has not been able to grasp the true nature of OCD patients’ suffering, with respect to their experiences of and representations of the world.

The present work seeks to consider the significance and potential contribution of a phenomenological reading of OCD and how phenomenalism has influenced cognitive models of this disorder. Phenomenology, according to its founder, Edmund Husserl [27], is an approach to philosophy that assigns primary importance to intuitive experience. Thus, it takes phenomena (as presented in phenomenological reflections that are indissolubly associated with the subject’s perspective) as starting points and attempts to extract from them their essential experiential characteristics. More precisely, this approach is known as “transcendental phenomenology”, and it has had a profound influence on modern cognitive frameworks.

## 3. The World of the Obsessive Patient: Phenomenological Considerations

Since the earliest descriptions of OCD, researchers have debated how the phenomenology of the disorder should be understood [28].

One of the first attempts to formulate an ‘explanatory’ model of OCD was proposed by Sigmund Freud [29,30]. Freud [31] certainly deserves merit for having considered obsessions and compulsions not as phenomena devoid of meaning, but as meaningful expressions that may be deciphered in light of the patient’s previous experiences—both recent and distant. However, over time, psychoanalysis has not proven effective in the treatment of OCD [32], and it may benefit from new theoretical approaches. For instance, object relations theory postulates a new obsessive position (as well as paranoid-schizoid and depressive positions) with respect to OCD [33], which may contribute significantly to psychoanalytic models. Additionally, the psychoanalytic approach to treating OCD may benefit from considering Kierkegaard’s notion of existential certainty, which is based on commitment, rather than evidence. In this perspective, the therapeutic role would be to support the OCD patient’s life concerns amid their tormenting doubts, without seeking resolution through the discovery of psychological truths [34].

Erwin Straus [35], one of the leading exponents of phenomenological psychiatry in the 20th century, noted that, every single day, obsessive patients are blocked by obstacles and impediments that do not constitute problems for “healthy” individuals. Straus brought attention back to the world of life, exploring themes of animality, corporeity, and sensory and motor experience, in order to deepen the preliminary and preconceived dimension of human and animal experience, beyond any idealization of traditional philosophy. His theory aimed at describing expressions of emotional life most appropriate to lived experience (where one’s experience of the world would be faithful to one’s intuitive experience), without giving preference to the intuitive level as something underlying and ‘more real’. Thus, his approach to phenomenological psychology underlined the importance of lived experience.

Straus’s systematic analysis laid the foundations for a new psychiatry enriched by patients’ lived experiences of OCD as a dialectical and communicative relationship with madness. These experiences were understood as both transcendental and material, a priori and corporal. Straus advocated that psychiatry should not attempt to define individual pathologies but should instead seek to understand the different ways of being in the world that characterize patients with different pathologies. With respect to OCD, he examined three clinical varieties of obsession: compulsive neurosis, contamination psychosis, and an intermediate variety called scrupulous neurosis. He described compulsive neurosis as a fight against the precise and identifiable instincts, which contrast with moral beliefs; and he described contamination psychosis—the most characteristic form of obsessive pathology—as authentic psychosis that might transition to schizophrenia.

This simple statement leads to a fundamental question that may help us to broaden our understanding of OCD: Why are ‘healthy’ individuals able to tolerate uncertainty, correctly assess danger, and attach proper importance to their thoughts, while OCD patients cannot [36,37,38,39]?

In attempting to answer this question, we will consider the traditional cognitive vision model [40], the post-rationalist cognitive model [41], and the neuropsychological cognitive psychotherapy approach [42,43].

More specifically, we will attempt to determine which of these models—all of which are influenced by phenomenological thought—can most effectively grasp the world that is experienced by OCD patients. 

According to traditional cognitive models [44,45,46], emotional disorders mainly consist of cognitive distortions that produce irrational or dysfunctional beliefs. Dysfunctional beliefs typical of OCD may include unrealistic beliefs about one’s ability to influence external events [47] and overestimations of responsibility (i.e., inflated responsibility) linked to harmless and ubiquitous thoughts [48]. 

Mental processes allow individuals to transform, reduce, store, and retrieve the information that reaches their sensory systems. Behavior is conceived of as a series of acts guided by cognitive processes to solve a problem, with continuous adjustment to ensure the best solution. The mind is therefore an apparatus with a fixed sequential organization that regulates behavior according to its momentary objectives and reduces the discrepancy between subjective representations and facts in “reality”.

For obsessive patients, the discrepancy between subjective experience and reality is so great that it leads to obsessions—that is, to incorrect assessments of the “truthfulness” of facts. OCS stems from patients’ incorrect thoughts and evaluations of themselves and external reality, as compulsions and rituals are introduced to reduce the negative emotions that arise from these dysfunctional assessments [49]. Several studies [50,51,52,53,54] have provided support for these cognitive hypotheses and helped to outline two fundamental concepts that may, from this perspective, contribute to OCD: feelings of guilt and inflated responsibility.

For obsessive patients, the possibility that one may be guilty of something is experienced as not only demeaning and negative (as for healthy people) but, above all, as an unforgivable catastrophe that cannot be overcome [55,56,57]. This self-consideration goes hand in hand with OCD patients’ exaggerated sense of responsibility [58,59], which Salkovskis and Forrester [60] defined as the belief that one has a fundamental role in determining outcomes and reducing negative consequences (i.e., through rituals or superstitious gestures).

According to cognitivism, uncertainty and doubt [61,62,63,64] are ill-tolerated by OCD patients, because an excessive sense of responsibility and guilt imprisons them in a vicious circle of thoughts (i.e., obsessions) and does not allow them to accept the situation as is. Their exaggerated and dysfunctional belief that they play a central role in determining consequences leads to an incorrect assessment of reality. Therefore, false, exaggerated, and wrong beliefs lie at the root of their condition.

Now, is it possible to understand a pathology as something that “happens” only in the mind? If one considers humans to be thinking machines, then this cognitive perspective is correct. However, this perspective, while contributing a meticulous and systematic analysis of the characteristics of obsessive thought, limits its observations to the functioning of the individual’s mind, while overlooking the fundamental sense and meaning of the world in which OCD patients are trapped.

Straus’s methodology of structural analysis [35] aims at tracing the constitutive essence and structure of psychic disorders. This involves comparing the characteristic elements of a given disorder with their correspondences in “normal” psychology. Straus claimed that, in psychiatric work, more attention should be paid to the standard modalities of phenomena, since it is only through comparison with “normality” that it is possible to achieve an adequate understanding of psychopathological manifestations. More specifically, he wrote [65] that “only after coming to understand the world in which the obsessive patient lives can one hope to know the genesis of the pathology”.

Straus’s phenomenological conception considered the individual an inhabitant of the human world. His theory aimed at describing and understanding the general expressions of emotional life that are most appropriate to experience, while consistently underlining the themes of animality and corporeity. Thus, lived experience took a central position in his approach.

Straus’s phenomenological goal was to describe an individual’s world in accordance with its manifest reality in a condition of ‘normality’. He theorized that pathology involves an interruption of the ‘normal’ subject-world relationship, and elaborated that, for OCD patients, the relationship with the world lacks continuity and form: existence becomes stripped of objects, moments, and sensations (i.e., it becomes a ‘shapeless whole’), and, lacking a sense of external organization, both objects, and the world lose their pleasantness and instead invoke emotions of disgust [35].

Post-rationalist cognitivism developed the cognitive model further, in harmony with the phenomenological approach. This model does not consider psychological reality objective and objectively definable, but instead regards it as the product of the interaction between the observer and the environment [66]. In this framework, the mind is not a simple passive computer of information. Rather, it builds (based on cognitive schemes that organize personal meaning) reality actively through interaction with—and the interpretation and classification of—the surrounding environment [41].

Self-consciousness, worldviews, and existential temporality, in narrative form, follow verbal, unconscious rules (i.e., tacit knowledge) [67]. The continuous assimilation of experience over time progressively increases the inner complexity of the self, taking the individual to more integrated levels of self and world knowledge [68]. 

Post-rationalist cognitivism identifies the basis of OCD not as cognitive distortions (i.e., unrealistic beliefs, overestimations of responsibility), but as a ‘personological configuration’, referring to a particular organization of personal meaning associated with obsession. While an obsessive personality configuration can also be found in normal subjects, it develops much more rigidly in OCD patients, thus predisposing them to psychopathological decompensation.

Guidano’s [69] dimensional framework describes a continuous spectrum of personological configuration between normality and psychopathology, comprised of two psychological dimensions. The first, which is already documented in the literature [70,71], is defined by the polarities of field dependence versus field independence. Field dependence describes the existence of interpersonal relationships and a relatively affective, contextual preference for interactive situations, while field independence describes a relatively impersonal cognitive approach and a lack of interest in the opinions of others. The second dimension, defined by the polarities of inwardness versus outwardness, describes a greater (i.e., inwardness) or lesser (i.e., outwardness) ability to focus on one’s experience. This dimension correlates with emotional regulation, with inwardness indicating a greater ability to experience a strong sensory load and basic, well-defined emotions, and outwardness indicating less univocal and defined emotional states, which require significant work to interpret.

Recently, a further dimension was proposed, characterized by the polarities of diachronic versus synchronic [72]. This dimension describes the need to perceive a continuity of experience (and ultimately self-image) over time (i.e., diachronicity) versus the need to perceive a unity of experience, moment by moment (i.e., synchronicity). In this framework, the ‘obsessive’ organization is characterized by outward, field-independent, and diachronic polarities. These are the epistemological criteria on which the post-rationalist psychotherapy of psychopathological disorders such as OCD is based [73].

Cognitive neuropsychological psychotherapy represents a natural evolution of cognitive psychotherapy. This approach combines recent developments in neuroscience, developmental psychology, and psychopathology into a unitary theoretical framework [74]. Through this rigorous and interdisciplinary perspective (referring to the phenomenological and hermeneutic traditions) [75], the individual represents an inseparable unity of mental processes, body, history, and planning. 

The dual belonging of the individual to both the natural and the psychological order can be configured as semantic dualism. The human sciences can dialogue with the biological sciences according to the paradigm of translation [76], considering psychology and neuroscience as two specialized languages that seek to say the same thing, using different terms.

Neuropsychology may provide a context for the interdisciplinary mediation between neuroscience and psychology. Accordingly, neuropsychopathology may benefit from a dialectic between physical causality and human motivation, considering that the latter is understandable only through the mediation of life history.

For etiopathogenetic purposes, the dual belonging to the physical and the psychic order allows us to configure a neuropsychopathological continuum in which fruitful dialogue is fundamental between the ‘bio’ and ‘psi’ disciplines. To this end, neuropsychology may play a vital role as a hermeneutic discipline.

This allows for psychotherapeutic interventions that are scientifically reliable and formalizable, yet not reduced to technical protocol. 

According to the cognitive-neuropsychological approach, vision and essence are not found in rationality or emotionality, as these aspects of being follow existence, but do not constitute it. Thus, any investigation of existence must be rooted in ways of being, understood as feelings of being situated and understanding. Feelings of being situated relate to the concept of belonging to the self of experience (i.e., ipseity). In turn, ipseity is always accompanied by a form of understanding the self-in-the-world, as a precondition for reflective reconfiguration.

Here, we would add to the phenomenological approach (which explains the prereflective function) the hermeneutical background, which explains the narrative nature of personal identity. According to Paul Ricoeur [77], personal identity is reconfigured according to new horizons, which are simultaneously phenomenological and hermeneutic, as the individual who acts and suffers is always located in a certain historical and cultural context.

If precomprehension of action–passion is the condition for an event to be spoken about, then language is the cultural device that allows for its narrative reconfiguration. On the one hand, action–passion is already significant within a context, while on the other hand, the story is only possible by means of language, which is historically and culturally located. Thus, the individual of phenomenology-hermeneutics is simultaneously embodied and historically embedded.

This model seems better able to grasp the meaning of the world of OCD patients, in alignment with Straus’s thesis that obsessive patients, defending themselves from the world and its “ugliness”, do nothing but isolate themselves and close themselves in their own reality, thus breaking the original relationships that characterize human beings. Through this lens, disgust is the meaning grasped at the world’s opening [43]. Therefore, the only conceivable mode of an obsessive relationship with the world is defense from it and its formless shape.

The phenomenological perspective, alongside the post-cognitive rationalist framework and neuropsychological cognitive psychotherapy, contributes significant insights into obsessive pathology by reflecting on the meanings of the world, as experienced by OCD patients.

The cognitive neuropsychological approach builds on Blankenburg’s [78] concept of natural evidence, related to the study of pauci-symptomatic psychosis. Natural evidence represents the background of pre-reflexive obviousness that gives continuity to experience (thematic consciousness), a sense of self, and temporality. It is altered in psychopathological situations, creating a feeling of detachment and unfamiliarity [79]. For OCD patients, their emotional sense of the world is insufficient. Therefore, their relationship to the environment must be managed and regulated rigorously and systematically, in reference to an external and abstract set of rules (undergoing continuous improvement). This allows them to maintain an adequate sense of personal stability [43]. OCD patients, therefore, meet each other and the world through a mediation of the reference system. OCS emerges from an alteration (i.e., “insecurity”) of personal identity due to a mismatch between the experience and the set of rules through which OCD patients perceive themselves [43,80]. Whenever an inconsistency occurs—that is, when experience can no longer be reconfigured through the set of rules—the subjective sensation is that of disintegration, since the sense of personal stability is shaken. Obsessions are the consequence of the interruption between experience and the reference system, and compulsions represent an attempt to reconnect these aspects through an immediate re-positioning [43].

For greater clarification, we present a clinical case in Table 1 explained both by the post-rationalist cognitivist approach and the neuropsychological approach.

According to the post-rationalist cognitivist approach, an individual with an obsessive personality configuration will experience a loss of control whenever they feel new emotions that are difficult to reconcile with those felt previously. Considering the field-independent psychological dimension, the above-described subject is likely to experience difficulty integrating different experiences into a unitary context. Indeed, his reaction consists of doubts about which state is ‘true’ (i.e., the current or the previous state). To regain self-control (a characteristic of the diachronic dimension), he will likely engage in a series of checks aimed at understanding who he really is and what he really feels.

According to the neuropsychological approach of cognitive psychotherapy, the subject will likely experience a highly historicized alteration of personal identity (affected by their OCD over prior years). He will not recognize an experiential state correlated with an unpredictable existential event (i.e., decreased interest due to his state of fatigue), and this failure to recognize an ‘identitarianly reconfigured’ experiential state will determine a reaction of doubt. The goal of psychotherapy should be to change his way of experiencing, in addition to modifying his way of describing his experience.

## 4. Conclusions

OCD remains a peculiar pathological condition, due to its paradoxical psychological and behavioral manifestations. While a descriptive psychopathology of OCD has long been available, to date, no satisfactory psychopathological model has been established.

Post-rationalist cognitivism [41], moving away from the traditional cognitivist model [40], recognizes in OCD a characteristic personological configuration (i.e., obsessive ‘organization of personal meaning’) that is exacerbated in OCD patients, in comparison to normal individuals. Additionally, neuropsychological cognitive psychotherapy [42,43], seeking to combine scientific rigor and progress with psychological knowledge, theorizes the concept of belonging to the self of experience (i.e., ipseity) as a necessary and indispensable element of understanding the self-in-the-world, which is a precondition for reflexive reconfiguration. Thus, the lack of recognition of an ‘identitarianly reconfigured’ experiential state may lie at the basis of OCD.

Both of these models, integrating phenomenology, lend themselves to exploring the particular relationship of OCD patients with their world, which is characterized by hyper-reflexivity, at the expense of natural evidence [78,79].

## Figures and Tables

**Table 1:** Clinical Case.

18-Year-Old Male Who Was Diagnosed with OCD Several Years Prior
The subject is passionate about natural history and assiduously watches a TV program on the subject. One evening, when he is particularly tired, he notices that he has little desire to watch his favorite program, and he considers this an ‘unequivocal and inexplicable’ drop in interest. He immediately asks himself what his ‘true’ state is: the current one (implying that he was previously deceived) or the previous one (implying that he is currently deceiving himself).

## Data Availability

Not applicable.
